# Dynamic changes in biomarkers in acute human immunodeficiency virus infections: a case report

**DOI:** 10.1186/s13104-017-2392-4

**Published:** 2017-01-26

**Authors:** Wei-Ming Gu, Yi Hu, Wei-Zhong Hu, Biao Xu

**Affiliations:** 1 Shanghai Dermatology Hospital, Shanghai, 200050 China; 2 School of Public Health, Fudan University, Shanghai, 200032 China

**Keywords:** HIV case report, p24 antigen, Antibody testing, Acute infection, Biomarkers, Men who have sex with men, China

## Abstract

**Background:**

The highest incidence of human immunodeficiency virus infection in China is among men who have sex with men. This case report aims to describe the dynamic changes in biomarkers in an acute human immunodeficiency virus infection of a Han Chinese man who has sex with men, and to illustrate the possibility of using these biomarkers for the early detection of human immunodeficiency virus infection in Chinese hospital settings.

**Case presentation:**

The 25-year-old Han Chinese male patient presented himself with an 8-day history of symptoms and signs of upper respiratory viral infections to a sexually transmitted infection clinic of a hospital setting in Shanghai. The viral load of human immunodeficiency virus, p24 antigen–antibody complex, and lymphocyte subsets of blood samples were repeatedly measured over the next 39 days. The human immunodeficiency virus from serum was genotyped. This patient was diagnosed as a human immunodeficiency virus infection, and the viral genotype was CRF 01_AE. The onset of the symptoms and signs was 12 days after his last reported unprotected intercourse with a human immunodeficiency virus -infected man. The patient had detectable levels of p24 antigen at his first visit, 20 days after infection, and the HIV viral load was at the highest point (8 × 10^6^ copies/ml). A low concentration of antibody to HIV was observed in the patient’s serum 10 days after his 1st visit (30 days after infection). The confirmation of human immunodeficiency virus infection by Western blot assays was made at day 20 after his 1st visit (40 days after infection).

**Conclusions:**

Symptoms of acute human immunodeficiency virus infection are non-specific. Specific laboratory markers appear shortly after HIV infections. The first biomarker detected from serum is the viral RNA and p24 antigen, followed by HIV-specific antibody. The results suggest that there are urgent needs for both human immunodeficiency virus antigen and antibody testing in routine medical practice, and that human immunodeficiency virus RNA testing should be recommended to detect early infection. Ethics approval was obtained from the Ethics Board of the Shanghai Dermatology Hospital.

## Background

By 2014, it was estimated that 0.78 (0.62–0.94) million people were living with human immunodeficiency virus (HIV) in China [1]. More than 90% of all reported cases were infected through sexual transmission [1]. While HIV prevalence among the general population in China remains low at 0.058%, early detection of HIV infection in high risk populations, such as men who have sex with men (MSM) and female sex workers (FSW), is of great importance [2, 3].

The timing of the appearance of viral and host markers in HIV infection has been well characterized [4]. It has been shown that the natural history of HIV infections remain the same no matter whether the virus is sexually transmitted or by the transmission route of intravenous inoculations [4]. The natural history of HIV infections can be divided into six stages. Viral RNA in plasma is the first detectable markers, followed by viral antigens, and then host specific antibodies [4]. This indicates that early diagnosis of acute HIV infection will rely on the detection of viral RNA and viral protein antigens.

Currently, there are three distinct testing paradigms of HIV testing in China, i.e. voluntary counseling and testing (VCT), high-risk group testing, and provider initiated testing and counseling (PITC) [5]. The first two are typically set in the public health system, specifically in the Centre for Disease Prevention and Control (CDC) and in the facilities for blood donation and family planning. In contrast, patients with primarily sexual health complaints, whether they are heterosexual or homosexual, commercial sex workers or not, the local clinics, especially clinics for sexually transmitted infections (STIs), are the most frequently visited health facilities for the diagnosis and treatment of HIV. When patients are seen by clinical facilities, they are more likely to get tested for HIV through PITC.

Currently in China, screening of HIV infection is mainly performed using antibody-based tests such as the enzyme-linked immunosorbent assays (ELISA), followed by confirmatory tests using the Western blot assays [6].

Unfortunately, risk-based screening for antibodies to HIV is usually not sensitive enough for early diagnosis, and may fail to identify HIV-infected individuals during the first several weeks following acquisition [7]. The improvement in the sensitivity of serologic tests (e.g., the HIV p24 antigen assay) [8], the detection of HIV viral load, and more recently the implementation of the HIV nucleic acid test (NAT) which can detect HIV RNA have all further reduced the delay in detecting acute infection [9–12]. HIV viral RNA is detectable approximately 7–11 days after HIV infection [4].

Currently in China’s hospitals, particularly in STI-specialized hospitals, there is more frequent use of antigen–antibody combination-based fourth generation HIV tests, which enhances the potential for timely detection of HIV infection in early stages. However, very little information on the early detection of acute HIV infection in China has been reported. Here we report a case of acute HIV infection in a MSM in Shanghai, China. Repeated measurements of the patient’s HIV viral load, p24 antigen–antibody complex, and lymphocyte subsets were taken. The aims of this case report are to describe the dynamic changes in these biomarkers in a patient with an acute HIV infection and to illustrate the possibility of detecting HIV infection in the window before the presence of HIV antibodies in clinical settings.

## Case presentation

### Demographic characteristics and history

Ethics approval was obtained from the Ethics Board of the Shanghai Dermatology Hospital. A 25-year old male Han Chinese patient presented himself to a STI clinic of a hospital in Shanghai. The patient had an 8-day-duration of a low fever (~38 °C), sore throat, periodontitis and torso rash before hospital admission. The patient was extremely worrying himself being infected with HIV. He had been engaging in MSM for about 8 years. He had met his latest male partner through internet 2 months ago and had several unprotected anal intercourses in the past 35 days. The last unprotected sex with his male partner occurred 10 days ago. The patient had received routine health check-up including HIV screening testing. The last health check-up was done 2 months ago, and HIV antibody testing was negative at that time. According to the HIV surveillance data in Shanghai Municipal CDC, the partner was confirmed with HIV infection 2 years ago; however, the patient had never been informed of his partner’s HIV status. The patient had unprotected anal intercourse with this MSM partner 10 days before the onset of the above symptoms and signs. The patient had no history of drug use or surgery. The patient was treated and currently is being followed up.

### Methods and results of laboratory testing

**Fig. 1:**
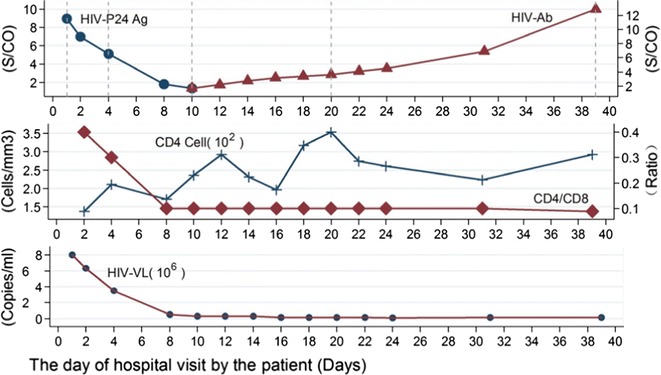
Dynamic changes of biomarkers in a HIV-infected patient. Day 0: the day of the patient’s first hospital visit. *S/CO* signal-to-cut-off, *HIV*-*p24 Ag* human immunodeficiency virus p24 antigen, *HIV*-*Ab* human immunodeficiency virus antibody, *HIV*-*VL* human immunodeficiency viral load

Blood samples of the patient were collected at the 1st visit on Day 1, 8 days after the onset of symptoms and signs, and at follow-up visits until Day 39 (Fig. 1). Biomarkers tested included HIV RNA load, HIV-p24 antigen, HIV antibody and blood lymphocyte CD4/CD8 counts and ratios. Briefly, the HIV RNA load was quantified by RT-PCR [Easy Q NASBA HIV-1 v2.0 (BioMérieux Clinical Diagnostics, Co., Ltd, Shanghai, China) and MyCycler™ thermal cycler (Bio-Rad Laboratories Co., Ltd, Shanghai, China)]. The HIV-p24 antigen was determined using VIDAS HIV DUO Ultra assay (BioMérieux Clinical Diagnostics, Co., Ltd). Confirmatory tests using the Western blot testing for HIV antibody was performed [HIV BLOT 2.2 MP diagnostics (MP Biomedicals Co., Ltd. Singapore)]. The HIV virus was also genotyped using the nested RT-PCR followed by DNA sequencing analysis [Clustal X program in MEGA5 software version 4.9, Center for Evolutionary Medicine and Informatics, The Biodesign Institute, Tempe, Arizona]. Drug-resistance associated mutations in protease and reverse transcriptase regions of the virus were also analyzed (Stanford University HIV/AIDS Drug Resistance Database; http://hivdb.stanford.edu/). The lymphocyte subsets were measured using the flow cytometry (Becton–Dickinson Medical Devices Co., Ltd., Shanghai).

HIV viral RNA was first detected on Day 1, about 20 days after the last incidence of unprotected intercourse and 8 days after the onset of symptoms and signs. The viral load on Day 1 was 8.0 × 10^6^ copies/ml. A sharp decrease in the viral load was observed between day 1 and day 16. The viral load after Day 16 remained was stable with approximately 1.0 × 10^5^ copies/ml (Fig. 1).

The HIV-p24 antigen was also first detected on day 1. The signal-to-cut-off (S/CO) values of p24 antigen declined quickly from 8.97 on day 1 to 1.35 on day 10. On day 12, the p24 antigen was no longer detectable in the serum.

HIV antibody in the serum was undetectable until day 10. The levels of HIV antibody titres rose after day 10, but remained low during the first 20 days of examinations. Western blot tests revealed only weak reactions in some of the bands during the same period and had a strong reaction on day 20.

The total counts of CD4^+^ T cells remained stable throughout the observation period. The percentage of CD4^+^ T cells was around 16% at the first two follow-up visits, and persisted at 6–8% from day 8 onward. The percentage of CD8^+^ cells underwent a sudden increase from 39% on day 2 to 69% on day 8, and persisted at this level through the follow-up period. The CD4^+^/CD8^+^ ratio varied from 0.4 on day 2 to 0.1 on day 8.

The HIV virus had a genotype of CRF 01_AE. There were no mutations in the resistant genes for drugs of nucleoside reverse transcriptase inhibitors, non-nucleoside reverse transcriptase inhibitors and protease inhibitors.

## Conclusions

The reported case is an acute HIV-CRF 01_AE infection in a Han Chinese male patient who had unprotected sex with a HIV infected man. The virus RNA and p24 biomarkers were detectable in the serum at early time of the infection. Viral RNA appeared high titre on the first day of visit. HIV-specific antibody was detectable after the appearance of HIV virus and HIV antigen. The dynamic changes in these biomarkers were consistent with the natural history and immune-pathogenesis of a typical HIV infection [4]. Most HIV screening tests in Chinese clinical settings detect antibodies but not viral antigen proteins or RNA [12, 13]. In the absence of antibodies, p24 antigen and RNA typically indicate acute HIV infection. Combined viral antigen and specific antibody may still miss HIV infections in acute stages (i.e. within 20 days after HIV acquisition) [4, 12]. This study suggests that detection of HIV virus RNA and HIV-p24 antigen in the serum are required for early diagnosis of HIV infections and recommends an urgent need for antigen–antibody combination-based HIV tests in clinical settings. In settings with appropriate facilities, HIV virus RNA testing should be recommended to detect early HIV infection.

## Abbreviations

HIV: human immunodeficiency virus
MSM: men who have sex with men
FSW: female sex worker
STI: sexually transmitted infection
VCT: voluntary counseling and testing
PITC: provider initiated testing and counseling
CDC: The Centre for Disease Prevention and Control (China)
ELISA: enzyme-linked immunosorbent assays
NAT: nucleic acid test
S/CO: signal-to-cut-off
HIV-p24 Ag: human immunodeficiency virus p24 antigen
HIV-Ab: human immunodeficiency virus antibody
HIV-VL: human immunodeficiency virus viral load
